# Capture and inactivation of viral particles from bioaerosols by electrostatic precipitation

**DOI:** 10.1016/j.isci.2023.107567

**Published:** 2023-08-09

**Authors:** Hannah E. Preston, Rebecca Bayliss, Nigel Temperton, Martin Mayora Neto, Jason Brewer, Alan L. Parker

**Affiliations:** 1 Division of Cancer and Genetics, Cardiff University School of Medicine, Heath Park, Cardiff CF14 4XN, UK; 2 Viral Pseudotype Unit, Medway School of Pharmacy, University of Kent, Central Avenue, Chatham ME4 4BF, UK; 3 Alesi Surgical Ltd, Medicentre, Heath Park Way, Cardiff CF14 4UJ, UK; 4 Systems Immunity University Research Institute, Cardiff University School of Medicine, Heath Park, Cardiff CF14 4XN, UK

**Keywords:** Virology, Particle Technology

## Abstract

Infectious viral particles in bioaerosols generated during laparoscopic surgery place staff and patients at significant risk of infection and contributed to the postponement of countless surgical procedures during the COVID-19 pandemic causing excess deaths. The implementation of devices that inactivate viral particles from bioaerosols aid in preventing nosocomial viral spread. We evaluated whether electrostatic precipitation (EP) is effective in capturing and inactivating aerosolized enveloped and non-enveloped viruses. Using a closed-system model mimicking release of bioaerosols during laparoscopic surgery, known concentrations of each virus were aerosolized, exposed to EP and collected for analysis. We demonstrate that both enveloped and non-enveloped viral particles were efficiently captured and inactivated by EP, which was enhanced by increasing the voltage to 10 kV or using two discharge electrodes together at 8 kV. This study highlights EP as an effective means for capturing and inactivating viral particles in bioaerosols, which may enable continued surgical procedures during future pandemics.

## Highlights

• Bioaerosols released from patients during surgery can facilitate viral spread
• Electrostatic precipitation captures and inactivates viral particles preventing spread
• Electrostatic precipitation is effective against enveloped and non-enveloped viruses
• Electrostatic precipitation represents a viable means to reduce nosocomial infections

## Graphical abstract

**Figure:**
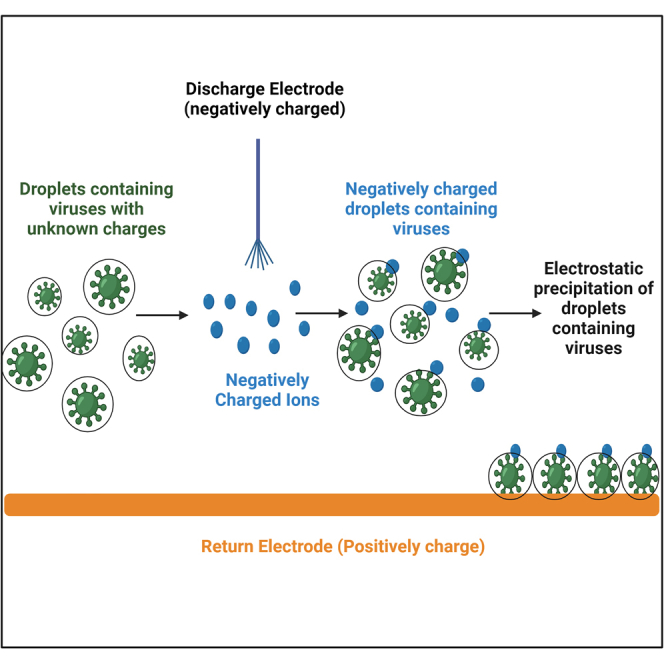

## Introduction

Acute respiratory viruses are the fourth leading cause of mortality worldwide.^1^ Although respiratory viruses can be spread by physical contact, contaminated fomites, and large droplets, key transmission occurs via the dispersion of bioaerosols from an infectious individual.^2^ Additionally, previous studies have shown that wild-type non-respiratory viruses, such as human immunodeficiency virus (HIV) and human papillomavirus (HPV) can also be released in bioaerosols, during aerosol-generating medical procedures, enabling viral transmission.^3^^,^^4^

With particular focus on the 2019 SARS-CoV-2 pandemic, >640 million cases and >6.5 million directly related deaths were reported worldwide in December 2022.^5^ Regarding the indirect consequences of the pandemic, it is estimated that hundreds of thousands of surgeries were delayed or canceled as a result. Bioaerosol-generating procedures, including laparoscopy, tracheostomy, open suctioning, and administration of nebulized treatments were at the highest risk of cancelation, due to the likelihood of airborne transmission to staff and other patients.^6^ This has left patients untreated and undiagnosed, creating enormous backlogs of waitlisted surgeries, thereby increasing the demand for private healthcare.^7^

Mitigation strategies such as mask wearing, personal protective equipment (PPE), social distancing, isolation of infected patients, and mass vaccinations were enforced and encouraged by the health authorities to reduce the spread of SARS-CoV-2.^8^ However, cases of SARS-CoV-2 infection continued to fluctuate at high levels, due to the evolution of new viral strains, easing of government-enforced restrictions and a lack in vaccine confidence by the general public.^9^^,^^10^ Therefore, the population remains at risk, emphasizing the need for novel non-pharmaceutical interventions (NPIs).

Commonly used NPIs for reducing the spread of disease in hospitals are ultra-low or high-efficiency particulate air filters (ULPA or HEPA), ultraviolet (UV) light sterilization, and aerosolized hydrogen peroxide (AHP) sprays.^11^^,^^12^ Although these NPIs are somewhat capable of purifying indoor air and decontaminating surfaces, each system is hindered by limitations. ULPA/HEPA filters are non-economical and labor intensive, as they use high levels of energy to run and require regular filter changes. Viruses that are trapped via a filter can remain live and active, adding an additional risk to their use within hospitals and requiring appropriate treatment as a biohazard during disposal.^13^ UV light is capable of inactivating viruses; however, its efficiency is limited to its alignment with and distance from the virus itself.^14^ As well as this, the exposure time and irradiance doses of UV light used to decontaminate indoor environments has not been well standardized, and incorrect usage of UV light can be hazardous.^14^ AHP sprays consist of 6% hydrogen peroxide mixed with 50 ppm silver ions and have been shown to eliminate SARS CoV-2 in nosocomial environments.^12^ Although AHP sprays are cost effective and have displayed efficacy as dry aerosol disinfectants, hydrogen peroxide is an irritant to the human skin and eyes, and if inhaled can be toxic.^15^

As nosocomial virus transmission occurs most commonly by the release of bioaerosols from infectious patients, it would be beneficial to develop an NPI that efficiently captures and inactivates viral particles from bioaerosols in hospital environments. Electrostatic precipitation (EP) technology has been developed to be used during key-hole surgeries, such as abdominal laparoscopies, to eliminate surgical smoke.^16^^,^^17^ Surgical smoke is produced by the thermal destruction of tissue by electrosurgical instruments during medical procedures and can obstruct the surgeons field of vision, resulting in safety implications.^18^ Surgical smoke consists of 95% water vapor and 5% cellular debris, of which can contain live bacterial and viral particles.^18^ EP clears surgical smoke via the generation of an electric field which precipitates particles out of aerosolized dispersion and onto a charged collection surface.^19^ This occurs by a discharge electrode emitting negatively charged ions into a neutrally charged space, creating a corona discharge.^20^ The current produced from a negatively charged discharge electrode results in the creation of low-energy gas ions and subsequent transient electrostatic charging of aerosolized matter within a local atmosphere. A return electrode carrying a positive charge is connected to a collector plate and located at a distance from the discharge electrode enabling the precipitation of negatively charged particles onto the positively charged collector plate via electrostatic attraction. This mechanism is exploited during key-hole surgery to clear surgical smoke, whereby aerosolized particles are ionized by a discharge electrode and precipitated onto the patient’s abdominal tissue, which is connected to a positively charged return electrode pad.^21^ Therefore, it was rational to assume that EP could also eliminate virus particles from surgical smoke, as bioaerosols released from patients consist of micrometer sized droplets, which can contain virus particles if the patient is infected. Subjecting virally contaminated aerosolized droplets to the negative charge emitted from the discharge electrode would thereby precipitate virus particles onto the positively charged return electrode, resulting in viral capture. Additionally, it was likely that EP could also inactivate virus particles from bioaerosols following contact with negatively charged air ions and formed radicals, as this has been previously suggested in other studies.^22^^,^^23^^,^^24^^,^^25^

It has been suggested that EP could be used in point-of-care systems as a method of aerosol sampling, to diagnose patients rapidly and accurately for respiratory viral infections, reducing the need to perform invasive and uncomfortable diagnostic procedures such as bronchoscopy.^26^ Furthermore, EP has been incorporated into a microfluidic lab-on-chip device, for immediate pathogenic detection from aerosol droplets released in the exhaled breath of patients.^26^ Custom bioaerosol samplers, employing EP mechanisms have also been developed and demonstrated to detect airborne influenza virus particles; of which studies have claimed may reduce sampling times down from hours to minutes, thus inhibiting viral transmission faster than currently existing approaches.^27^ EP is thereby capable of efficiently capturing airborne virus particles. Besides medical applications, EP has been used for decades in aerosol science to collect aerosol particles onto substrates for subsequent morphological analysis by scanning electron microscopy (SEM) and total reflection x-ray fluorescence (TXRF).^28^^,^^29^

Since EP is capable of efficiently clearing surgical smoke and has the capacity to capture airborne virus particles, it was rational to evaluate the ability of EP to capture and inactivate aerosolized viral particles from bioaerosols. Furthermore, EP has already been cleared by regulators as safe and effective in use,^16^^,^^30^ thereby serving as a practical, multi-modal device to use during medical procedures to prevent the spread of aerosolized viral particles. In addition, EP is capable of precipitating particles at a minimum diameter of 7 nm,^17^ thus improving the efficiency of particle capture and filtration compared to other established and commonly used ventilation and filtration systems, providing an alternative NPI for reducing disease transmission in hospitals.

The objective of our study was to evaluate the capture and inactivation of bioaerosol-containing viral particles by EP. Non-enveloped (Ad5) and enveloped (SARS-CoV-2 pseudotyped lentivirus) viral particles were aerosolized into a closed-system model, that was representative of key-hole surgery, and exposed to EP. Recovered samples were analyzed for viral presence by real-time quantitative polymerase chain reaction (qPCR) of viral genomes and for biological activity by transduction and plaque assays in target cell lines. We hypothesized that viral exposure to EP would result in significant viral capture and inactivation.

Reducing viral transmission is not limited to SARS-CoV-2, but accounts for all viral outbreaks that may lead to future pandemics. It is therefore important that novel NPIs are evaluated and developed, to increase our preparation, improve safety within hospitals, and prevent the need to cancel surgeries and medical procedures in the case of future pandemics.

## Results

### Ad5 particles were successfully captured and inactivated by electrostatic precipitation when aerosolized at 37°C

**Figure 1:**
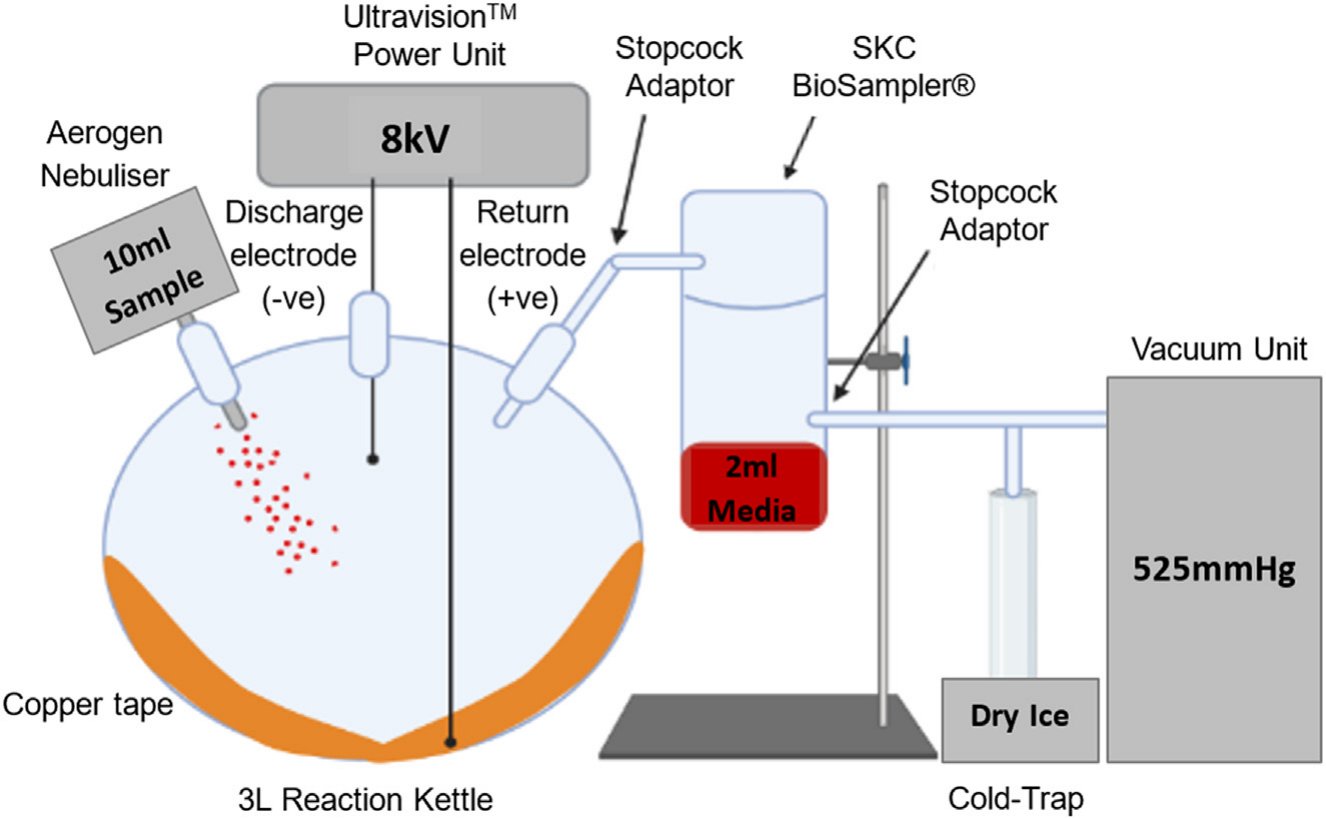
Schematic of the experimental setup of the refined closed-system model All samples were aerosolized into the air-tight reaction kettle, exposed to EP (active/inactive) and suctioned into the BioSampler for recovery and collection. Collected samples were stored at −80°C immediately after each experimental run, prior to experimental analysis.

**Figure 2:**
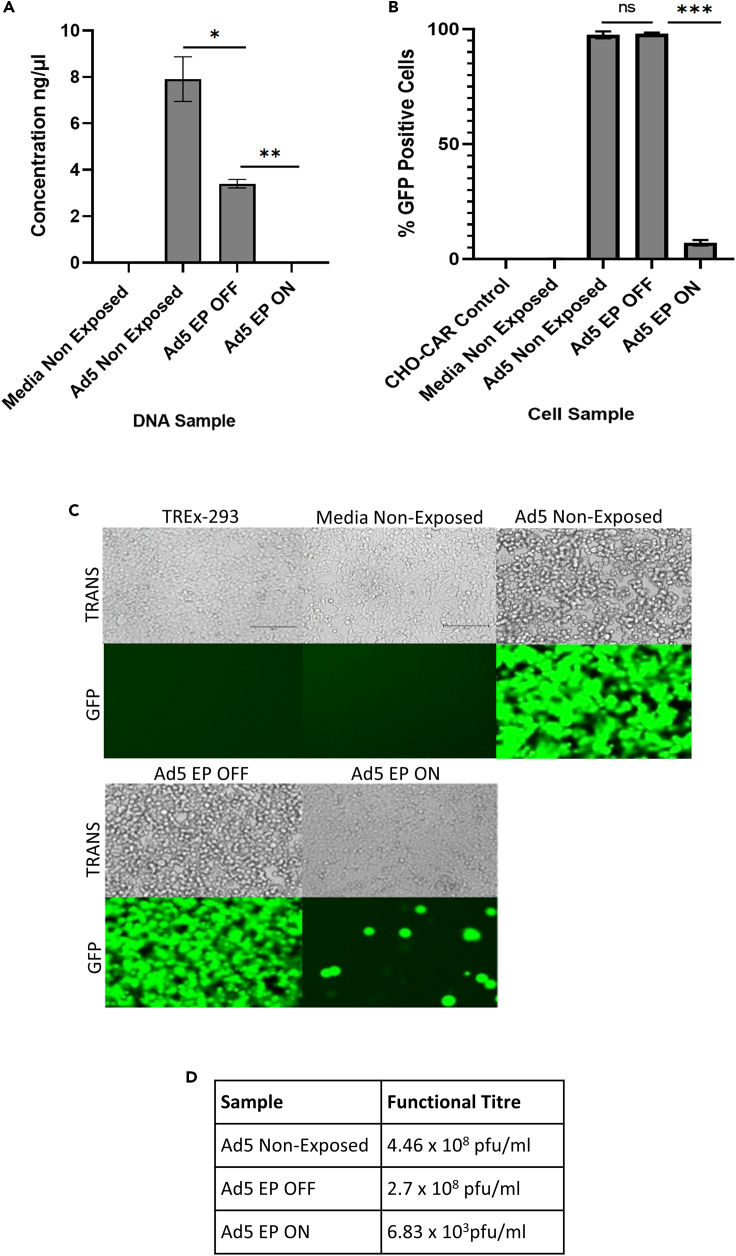
Capture and inactivation of Ad5 by electrostatic precipitation “EP OFF” signifies sample exposure to inactive EP and “EP ON” signifies sample exposure to active EP. “Non-Exposed” signifies samples that were not aerosolized through the model system, nor exposed to EP. (A) Viral capture quantified by qPCR. (B) Viral inactivation demonstrated by transduction assay. (C and D) Viral inactivation displayed by plaque assay in TREx-293 cells. TREx-293 cells treated with samples and analyzed for GFP fluorescence. TRANS = Brightfield transmitted light, GFP = GFP light source. Error bars represent the ±SD (n = 3). Plaque assay functional titers represent the mean (n = 5). Significance values represent ∗p < 0.05, ∗∗p < 0.005, ∗∗∗p < 0.0005.

First, we sought to evaluate whether EP could capture and inactivate aerosolized non-enveloped Ad5 particles using our standard closed-system model (shown schematically in Figure 1). The number of recovered Ad5 genomes significantly decreased following Ad5 exposure to inactive EP as gauged by qPCR for viral genomes, indicating viral loss as a result of sample aerosolization alone (Figure 2). A significant 6.8-fold reduction in the number of recovered Ad5 genomes was observed following Ad5 exposure to active EP (Figure 2A). Ad5 viability was not affected following exposure to inactive EP, as displayed by transduction and plaque assays (Figures 2B and 2C), indicating that sample aerosolization at 37°C was not detrimental to Ad5. The transduction assay demonstrated a 13.6-fold reduction in the percentage of transduction, in cells that were treated with Ad5 that had been exposed to active EP (Figure 2B). Mirroring this, the plaque assay displayed a 4x10^3^-fold reduction in active Ad5 particles, in the sample exposed to active EP (Figures 2C and 2D). These results indicated that EP successfully captured and inactivated aerosolized Ad5 particles within our standard closed-system model.

### Capture and inactivation of Ad5.GFP was most efficient when exposing viral particles to 10kV

Multiple parameters may impact the efficiency of EP. We assessed the impact of increasing voltages on the ability of EP to capture and inactivate aerosolized Ad5. EP is currently used at 8 kV to clear surgical smoke during laparoscopies. We exposed aerosolized samples of Ad5 to EP active at 6 kV, 8 kV, and 10 kV, to determine whether decreasing or increasing the standard voltage impacted its ability to capture and inactivate viral particles. By increasing the voltage of EP, the region of corona discharge was expanded, thus reaching a larger surface area and contacting more aerosolized virus particles. As 10 kV is the maximum voltage that is medically approved for EP use during surgery, voltages above this were not evaluated.

**Figure 3:**
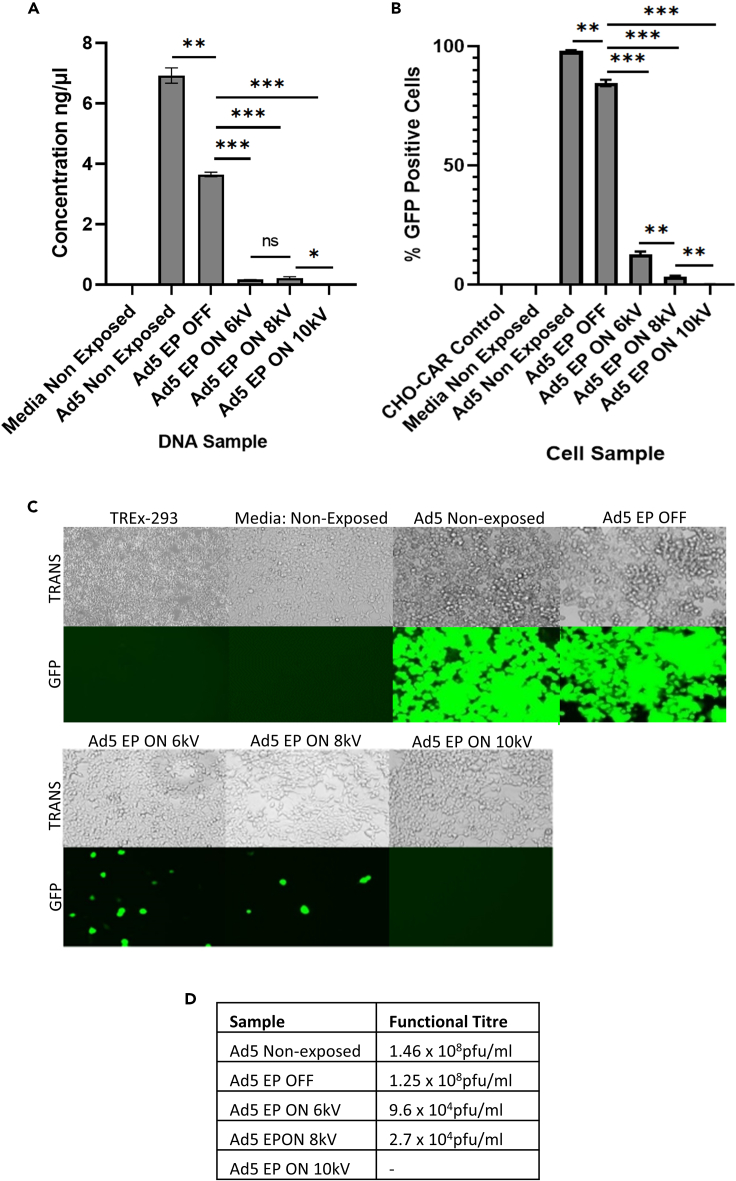
Increasing the voltage of EP to 10 kV enhances viral capture and inactivation “EP OFF” signifies sample exposure to inactive EP and “EP ON” signifies sample exposure to active EP. “Non-Exposed” signifies samples that were not aerosolized through the model system, nor exposed to EP. (A) Viral capture demonstrated by qPCR. (B) Viral inactivation determined by transduction assay. (C and D) Viral inactivation displayed by plaque assay in TREx-293 cells. TREx-293 cells treated with samples and analyzed for GFP fluorescence. TRANS = Brightfield transmitted light, GFP = GFP light source. Error bars represent the ±SD (n = 3). Plaque assay functional titers represent the mean (n = 5). Significance values represent ∗p < 0.05, ∗∗p < 0.005, ∗∗∗p < 0.0005.

qPCR analysis of treated samples indicated significant viral capture by EP, following sample exposure to 6 kV, 8 kV, and 10 kV (Figure 3A). The number of viral genomes was reduced by 21.8-fold and 16.8-fold, following Ad5 exposure to 6 kV and 8 kV, respectively. However, Ad5 capture was enhanced when exposing the viral particles to 10 kV, as shown by a 7.4x10^3^-fold reduction in the number of viral genomes (Figure 3A). Increasing the voltage to 10 kV also improved viral inactivation, demonstrated by transduction and plaque assay (Figures 3B and 3C). The percentage of transduced cells infected with Ad5 samples that had been exposed to 6 kV and 8 kV was significantly reduced by 6.6-fold and 25.6-fold, respectively (Figure 3B). Cells treated with Ad5 that had been exposed to 10 kV displayed a 529.4-fold reduction in viral transduction (Figure 3B). Mirroring this, plaque assays of treated samples demonstrated a significant decrease in the number of viable Ad5 particles in samples that were exposed to 6 kV, 8 kV, and 10kV (Figure 3C and D). Imagining of GFP highlighted a complete absence of viable Ad5 particles in cells infected with Ad5 samples that had been exposed to 10 kV, indicating that 10 kV is the optimal voltage to elicit efficient EP of bioaerosols during surgery, to completely prevent the transmission of infectious aerosolized virus particles (Figure 3C). While 6 kV significantly reduced the number of viable virus particles, EP by 8 kV and 10 kV resulted in log reductions of >3.5, suggesting a decrease within a clinically significant range.

### Using 2 discharge electrodes enhanced adenoviral capture and inactivation

We next evaluated whether enhanced viral inactivation was possible when exposing aerosolized Ad5 particles to 2, rather than a single discharge electrode. Both discharge electrodes were used at 8 kV, maintaining the voltage setting that is currently used during laparoscopic surgery. Separate Ad5 samples were exposed to either 1 or 2 discharge electrodes, to evaluate whether combining 2 discharge electrodes improved viral capture and inactivation.

**Figure 4:**
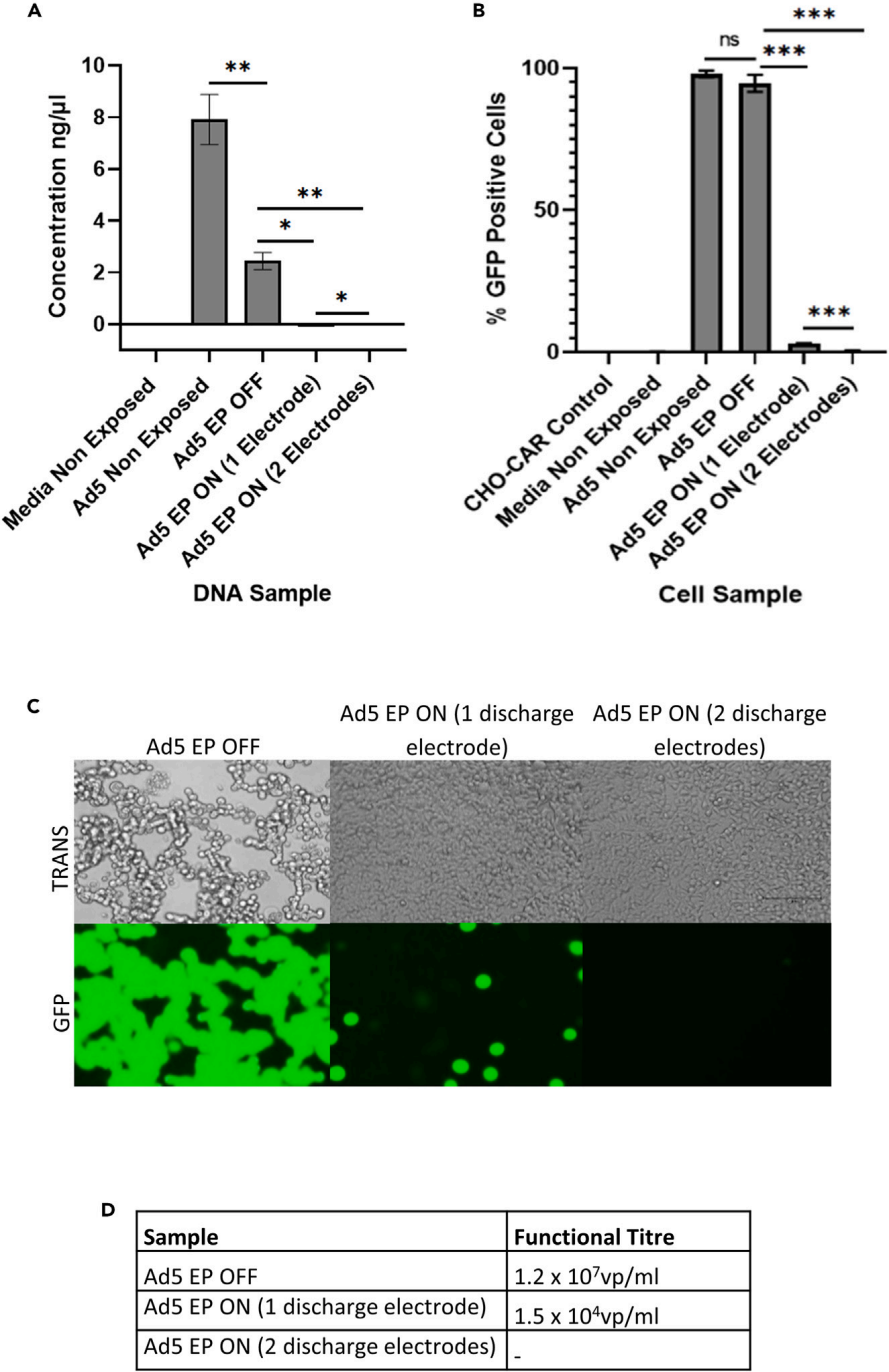
Exposing Ad5 particles to 2 discharge electrodes, opposed to 1, enhances viral capture and inactivation “EP OFF” signifies sample exposure to inactive EP and “EP ON” signifies sample exposure to active EP. “Non-Exposed” signifies samples that were not aerosolized through the model system, nor exposed to EP. (A) Viral capture demonstrated by qPCR. (B) Viral inactivation determined by transduction assay. (C and D) Viral inactivation displayed by plaque assay in TREx-293 cells. TREx-293 cells treated with samples and analyzed for GFP fluorescence. TRANS = Brightfield transmitted light, GFP = GFP light source. Error bars represent the ±SD (n = 3). Plaque assay functional titers represent the mean (n = 5).. Significance values represent ∗p < 0.05, ∗∗p < 0.005, ∗∗∗p < 0.0005.

qPCR results displayed a significant decrease in the number of viral genomes in Ad5 samples that were exposed to either 1 or 2 active discharge electrodes. A 125-fold reduction in the number of Ad5 genomes was observed in the sample exposed to 1 active discharge electrode, whereas exposure of Ad5 to 2 discharge electrodes resulted in an increased 1.25x10^3^-fold reduction in the number of Ad5 genomes detected (Figure 4A). This indicated that using 2 discharge electrodes, both active at 8 kV, enhanced viral capture by a further 10-fold. Similarly, Ad5 samples exposed to 1 or 2 discharge electrodes were both significantly inactivated. Cells treated with the Ad5 sample that had been exposed to a single active discharge electrode displayed a 31.6-fold reduction in the percentage of virally transduced cells (Figure 4B). In comparison, cells treated with the Ad5 sample that had been exposed to 2 active discharge electrodes displayed a 215.2-fold reduction in the percentage of transduced cells, indicating that using 2 discharge electrodes enhanced viral capture (Figure 4B). Plaque assay confirmed these findings, as shown by an 800-fold decrease in the number of active Ad5 particles, post exposure to a single discharge electrode, in comparison to a complete elimination of active Ad5 particles, post exposure to 2 discharge electrodes (Figures 4C and 4D). This experimental run highlighted that using 2 discharge electrodes enhanced viral capture and inactivation in a synergistic manner.

### Replacing the copper return electrode with a stainless-steel electrode indicated that electrostatic precipitation was the sole cause of viral inactivation

In previous runs, copper tape was attached to the positively charged return electrode, functioning as a collector plate for the precipitation of ionized virus particles. However, copper is a naturally virucidal metal and studies have shown direct contact between copper and viral particles resulting in viral inactivation.^31^ Therefore, we hypothesized that direct contact between the aerosolized viral particles and the copper tape may have been causing the viral inactivation observed in previous runs. To determine whether EP or the copper tape was causing viral inactivation, stainless-steel sheets were used to replace the copper tape. Stainless-steel is a biologically inert, non-toxic metal,^32^ and should not inactivate Ad5 particles upon direct contact. Ad5 samples that were not aerosolized, nor exposed to EP, were exposed to the stainless-steel sheets (direct contact for 2 min) and analyzed for viral activity in the same way as the collected experimental samples.

**Figure 5:**
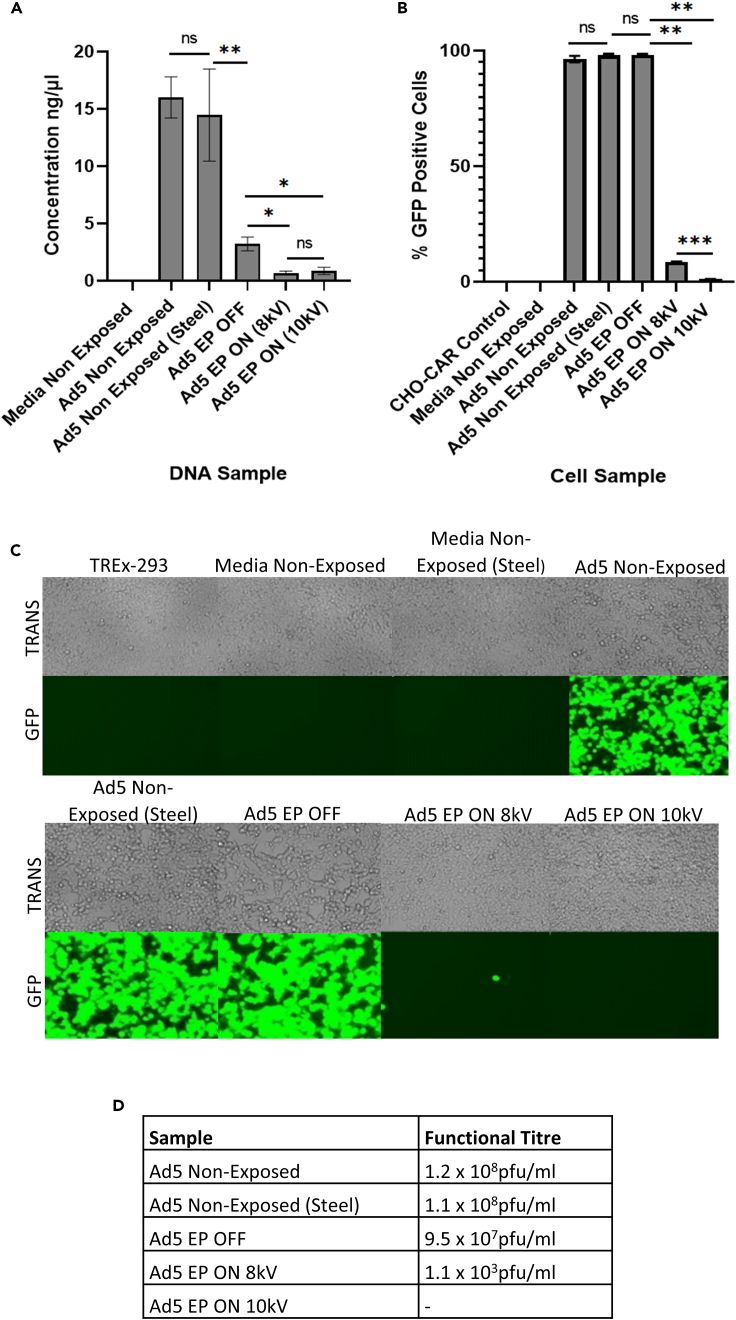
Evidencing EP as the sole cause of viral inactivation “EP OFF” signifies sample exposure to inactive EP and “EP ON” signifies sample exposure to active EP. “Non-Exposed” signifies samples that were not aerosolized through the model system, nor exposed to EP. “Steel” signifies samples that were exposed (direct contact) to stainless-steel for 2 min. (A) Viral capture demonstrated by qPCR. (B) Viral inactivation determined by transduction assay. (C and D) Viral inactivation displayed by plaque assay in TREx-293 cells. TREx-293 cells treated with samples and analyzed for GFP fluorescence. TRANS = Brightfield transmitted light, GFP = GFP light source. Error bars represent the ±SD (n = 3). Plaque assay functional titers represent the mean (n = 5). Significance values represent ∗p < 0.05, ∗∗p < 0.005, ∗∗∗p < 0.0005.

There was no significant difference between the number of Ad5 viral genomes in the non-exposed Ad5 sample and the Ad5 sample that was exposed to stainless-steel (Figure 5A). This indicated that stainless-steel did not alter the integrity of the viral DNA. The number of Ad5 genomes was significantly decreased in the Ad5 sample exposed to inactive EP, indicating that aerosolization alone resulted in a reduction in viral DNA collected within the sampling system, or potentially highlighting a size-specific particle loss phenomenon. However, the number of viral genomes was further significantly reduced in Ad5 samples following exposure to active EP at 8 kV and 10 kV (Figure 5A). This indicated that EP successfully captured the aerosolized Ad5 particles. Cells treated with non-exposed Ad5 and the Ad5 sample that was non-exposed to the closed-system but exposed to stainless-steel showed no significant difference in the percentage of virally transduced cells (Figure 5B). Plaque assay results mirrored this result, showing no visible differences between TREx-293T cells infected with either sample (Figure 5C). This indicated that direct contact between Ad5 particles and stainless-steel did not affect viral viability. In addition, CHO-CAR cells infected with Ad5 samples exposed to active EP at 8 kV and 10 kV displayed 11.32-fold and 86.9-fold reductions in the percentage of virally transduced cells, indicating successful inactivation of Ad5 particles by EP (Figure 5B). Confirming this, TREx-293T cells infected with Ad5 samples that had been exposed to active EP at 8 kV and 10 kV showed visibly reduced levels of fluorescence, indicating successful inactivation (Figure 5C).

### Electrostatic precipitation successfully captured and inactivated enveloped viral particles (SARS-2 PV)

Finally, we sought to evaluate the ability of EP to capture and inactivate enveloped viral particles, such as SARS-CoV-2. As Ad5 is a non-enveloped virus, we used a SARS-CoV-2 pseudotyped lentivirus (SARS-2 PV), as its core and genetic material is enclosed by a lipid envelope which expresses the Wuhan Spike protein on its surface, thereby resembling the external structure of wild-type SARS-CoV-2. Neat samples of SARS-2 PV were aerosolized and exposed to EP in the same way as Ad5 in Figure 1.

**Figure 6:**
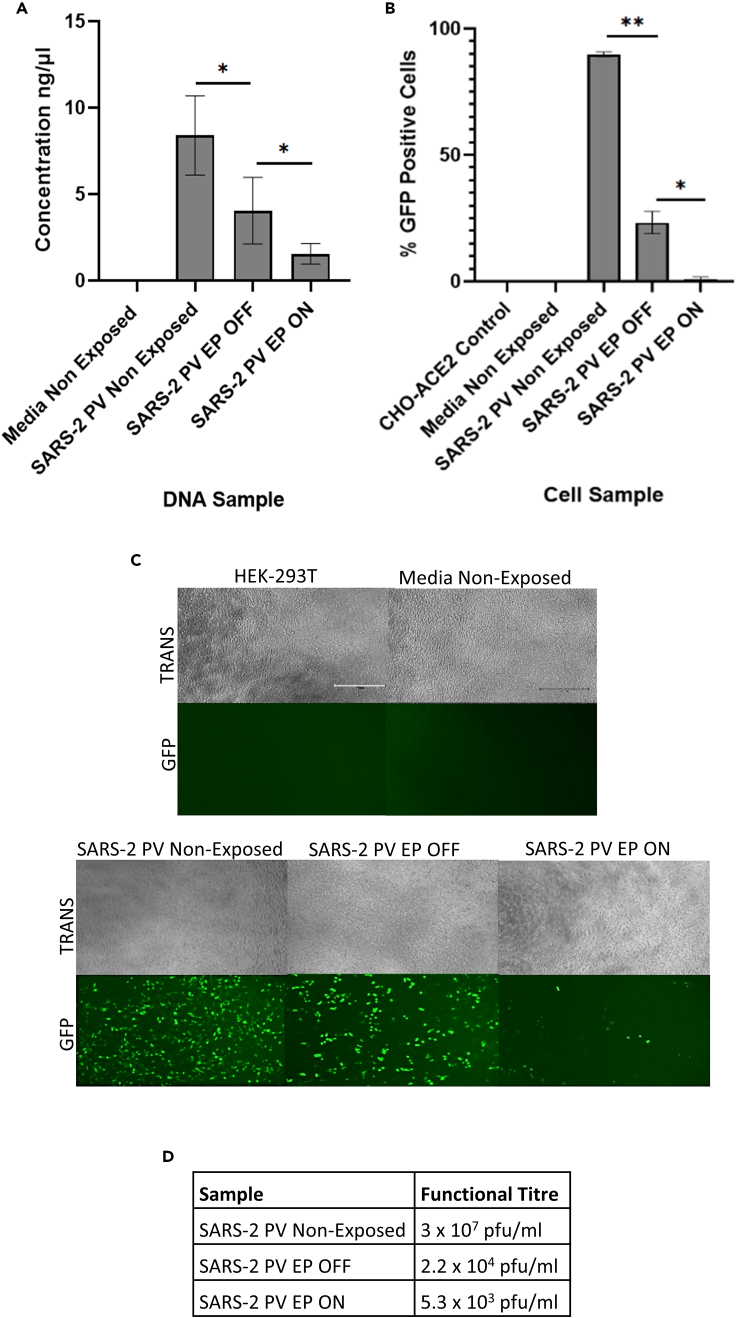
Capture and inactivation of SARS-2 PV by EP “EP OFF” signifies sample exposure to inactive EP and “EP ON” signifies sample exposure to active EP. “Non-Exposed” signifies samples that were not aerosolized through the model system, nor exposed to EP. (A) Viral capture determined by qPCR. (B) Viral inactivation demonstrated by transduction assay. (C and D) Viral inactivation displayed by plaque assay in HEK-293T cells. HEK-293T cells treated with samples and analyzed for GFP fluorescence. TRANS = Brightfield transmitted light, GFP = GFP light source. Error bars represent the ±SD (n = 3). Plaque assay functional titers represent the mean (n = 5). Significance values represent ∗p < 0.05, ∗∗p < 0.005.

SARS-2 PV was significantly captured by EP, as quantified by qPCR (Figure 6A). A 2.6-fold reduction in the number of viral genomes was observed in the SARS-2 PV sample that had been exposed to active EP, indicating successful virus capture (Figure 6A). In addition, transduction and plaque assays using the collected samples showed that EP significantly inactivated aerosolized SARS-2 PV particles (Figures 6B–6D). CHO-ACE2-TMPRSS2 cells infected with the SARS-2 PV sample that had been exposed to active EP displayed a 27.7-fold reduction in the percentage of viral transduction (Figure 6B). Likewise, HEK-293T cells infected with SARS-2 PV that had been exposed to active EP displayed a visually decreased number of fluorescent cells, compared to the non-exposed sample and the SARS-2 PV sample exposed to inactive EP (Figure 6C). However, the number of viral genomes, as well as viral viability, was significantly reduced in the SARS-2 PV samples that were aerosolized and exposed to inactive EP (Figure 6). This indicated that aerosolized SARS-2 PV was less stable than aerosolized Ad5, and that the sample was more susceptible to inactivation or degradation by aerosolization alone.

## Discussion

Existing methods of purifying indoor air are limited by their inability to capture aerosolized particles smaller than 0.15 μm and failure to inactivate live pathogens upon successful capture.^13^ These limitations facilitate disease transmission. During periods of viral outbreaks, such as the 2019 SARS-CoV-2 pandemic, bioaerosol-generating medical procedures are at risk of cancellation and delay, due to the likelihood of viral spread.^6^ It is therefore crucial that novel NPIs are developed to prevent airborne viral transmission in hospital settings, enabling medical procedures to continue safely and as normal. Established EP systems are currently used to sample and filter indoor air, as well as to clear surgical smoke during key-hole surgeries. Here we have demonstrated additional modalities of EP, in its ability to efficiently capture and inactivate aerosolized viral particles.

Significant capture and inactivation of aerosolized Ad5 and SARS-2 PV particles by EP was observed in our standardized closed-system model. Viral capture was displayed by a reduction in the number of viral genomes collected within the sampling system, following sample exposure to active EP, compared to recovered samples exposed to inactive EP. Similarly, viral inactivation was shown by a reduction in biological activity of viral particles, as gauged by the percentage of transduced cells that were treated with recovered samples post exposure to active EP, compared to samples exposed to inactive EP. Interestingly, it appeared that viral inactivation by EP was more successful than viral capture. Although the copper collector plate used within our closed-system model was naturally virucidal, our findings show that EP was the major cause of viral inactivation. However, using a virucidal collector plate, such as copper, may provide additional safety benefits for the removal of viable pathogens from bioaerosols by EP, thereby outperforming existing devices like HEPA filters.

Viral inactivation by EP was highly efficient, at approximately 90–95% efficiency when using EP at 8 kV, and at >99% efficiency when using EP at 10 kV or when using 2 discharge electrodes (both at 8 kV). Arguably, viral inactivation is more important than viral capture, as this can prevent the spread of disease. Previous studies evaluating the ability of EP to inactivate viruses suggest that the corona discharge, produced by the discharge electrode, generates air ions and reactive species (O_3_ and various radicals, such as O·, N·, OH·, and HO_2_·) capable of degrading and inactivating viral particles.^22^^,^^23^^,^^24^^,^^25^ Although this mechanism has not been explicitly investigated here, our results indicate that this could be the cause of viral inactivation. In agreement, degradation of viral particles would result in the release of viral DNA/RNA, explaining the collection of viral genomes in the sampling system following sample exposure to active EP. As isolated viral DNA is biochemically inert and requires an intact capsid to bind and enter target cells, the degradation of aerosolized viral particles seems a practical way of inactivating viruses and reducing their transmission.^33^^,^^34^

We have demonstrated that EP can efficiently capture and inactivate both non-enveloped (Ad5) and enveloped (SARS-2 PV) viral particles. However, aerosolization alone significantly reduced SARS-2 PV viability and the integrity of its capsid, causing the release of its viral genome. This was not surprising as SARS-2 PV is not a respiratory virus and is therefore not transmissible via airborne routes. However, other non-respiratory viruses, such as wild-type HIV and HPV, have been identified in surgical bioaerosols with the ability to infect healthcare staff. Therefore, it is important that EP can capture and inactivate a variety of viral particles.^3^^,^^4^ Future studies will focus on evaluating the ability of EP to capture and inactivate respiratory enveloped viruses, as well as non-respiratory non-enveloped viruses. In addition, other physical parameters govern viral spread and stability, including temperature, humidity, droplet size, and air-space volume.^35^ Evaluating changes to viral capture and inactivation, following the alteration of such parameters, as well as parameters effecting the efficiency of EP, such as voltage, flow rate, geometric design of the EP system, and size and concentration of the ionized particles,^36^ will be important to optimize in future studies, prior to implementing EP in hospitals as a method of reducing viral spread.

In addition, EP may play a role beyond clearing surgical smoke and eliminating viral particles during key-hole surgery. Due to recent advances in EP technology, it is likely that EP will be employed during open surgeries in the near future to clear surgical smoke. It is therefore possible that EP could be manipulated to capture and inactivate viral particles in “open” systems. For example, EP could be used to filter the release of CO_2_ upon patient deflation following laparoscopic surgery, as well as during open surgery, to filter bioaerosols released into the surgical environment in an attempt to protect healthcare professionals within close proximity. This could provide an alternative and intriguing means of replacing HEPA filters, which are currently used to filter bioaerosols in open environments. However, this would of course require adaptations to the device itself to enable sufficient exposure of the corona discharge to bioaerosols covering a much larger surface area succeeding release from the patient. As well as this, EP could be implemented when delivering aerosolized medications or advanced therapy medicinal products (ATMPs) to patients. For example, pressurized intraperitoneal aerosol chemotherapy (PIPAC) has recently been developed as a method of treating unresectable metastatic peritoneal tumors.^37^^,^^38^ PIPAC is an emerging technology and may be useful for more novel therapeutic deliveries, such as oncolytic virotherapies. Moving forwards, use of these technologies will require efficient means of controlling their emission during delivery. EP could be implemented during this type of therapeutic delivery to prevent the escape of oncolytic viruses into operating theaters, while simultaneously ensuring and directing efficient delivery of drugs to the tumor site. PIPAC has been developed for use during key-hole closed surgery; therefore, EP could be placed within the patient’s abdomen for the duration of drug delivery, as it already is during abdominal laparoscopies that use EP to clear surgical smoke.

In summary, our findings indicate that EP could be used during surgery to capture and inactivate viral particles released in bioaerosols, as well as potentially during other medical procedures, to enhance efficacy and safety. Employing EP as an NPI to reduce viral spread in hospitals may resolve issues experienced with existing air-purification systems, which in turn could reduce pressures on the NHS by preventing indirect morbidities and mortalities. For example, recent outbreaks of the highly pathogenic avian influenza A (H5N1) in wild birds and poultry has the capacity to spread to human hosts, which if unprevented, could result in the next human global pandemic.^39^ Using data obtained from this study, we predict that it is possible to use EP to minimize viral spread thus preventing future viral pandemics.

## STAR★Methods

### Key resources table

**Table:** 

REAGENT or RESOURCE	SOURCE	IDENTIFIER
**Bacterial and virus strains**		

Ad5.GFP	In-house (Stanton et al.^40^)	N/A
SARS-2 PV	(Di Genova et al.^41^)	N/A

**Chemicals, peptides, and recombinant proteins**		

Caesium Chloride	Invitrogen™	15507-023
0.45 μm acetate cellulose filter	StarLab	E4780-1453
FuGene® HD Transfection reagent	Promega	E2311

**Critical commercial assays**		

Micro BCA™ Protein Assay Kit	Thermo Fisher	23235
QIAamp MinElute Virus Kit	Qiagen	57704
PowerUp SYBR Green Master Mix	Thermo Fisher	A25741

**Deposited data**		

Raw and analyzed data	Mendeley Data Repository	Access numbers required

**Experimental models: Cell lines**		

Human T-REx-293	Invitrogen™	R71007
Human HEK-293T/17 cells	ATCC	CRL-1573
Hamster CHO	ATCC	
Hamster CHO-CAR	(Uusi-Kerttula et al.^42^)	N/A
Hamster CHO-ACE2-TMPRSS2	(Rebendenne et al.^43^)	N/A

**Oligonucleotides**		

Primers Ad5 Hexon - Forward: CCTGCTTACCCCCAACGAGTTTGA. Reverse: GGAGTACATGCGGTCCTTGTAGCTC.	Thermo Fisher	N/A
Primers P24 Capsid – Forward: GGCTTTCAGCCCAGAAGTGATACC. Reverse: GGGTCCTCCTACTCCCTGACATG.	Thermo Fisher	N/A

**Recombinant DNA**		

Spike SARS2 (D614G)-pCAGGS	NIBSC	CFAR100985
pCSGW encoding Green Fluorescent Protein	(Carnell et al.^44^)	N/A
Lentiviral Core p8.91	(Carnell et al.^44^)	N/A
MT126 pRRL- SFFV-ACE2-IRES plasmid	AddGene	145839
MT131 pRRL- SFFV-TMPRSS2.v1-IRES plasmid	AddGene	145843

**Software and algorithms**		

QuantStudio™ 5 Real-Time PCR	Thermo Fisher	https://www.thermofisher.com/uk/en/home/global/forms/life-science/quantstudio-3-5-software.html
FlowJo™v10	BD Biosciences	https://www.flowjo.com/solutions/flowjo/downloads
Prism v4.03	GraphPad	https://www.graphpad.com/scientific-software/prism

**Other**		

Aerogen® Solo Nebuliser	Aerogen Ltd	AG-A53000-XX
QuickFit™ Wide Neck Flask Reaction 3L	Scientific Laboratory Supplies Ltd	QFR3LF
QuickFit™ Borosilicate Glass Flange Lid	Fisher Scientific	MAF3/52
Ultravision™ Generator	BOWA Medial UK	DAD-001-015
Ionwand™	BOWA Medial UK	DAD-001-003
Suba-Seal®	Sigma-Aldrich	Z124621
QuickFit™ Borosilicate Glass Stopcock Adaptors	Fisher Scientific	MF14/3/SC
Duet Flat- Back Aspirator	SSCOR	2314B
BioSampler®	SKC Ltd	225-9595
QuickFit™ Cold-trap	VWR	201-3052
NanoSight NS300	Malvern Panalytical	N/A
EVOS M7000	Invitrogen™	AMF7000
Accuri C6 v.1.0.264.21	BD Biosciences	N/A

### Resource availability

#### Lead contact

Further information and any related requests should be directed to and will be fulfilled by the lead contact, Professor Alan Parker (ParkerAL@cardiff.ac.uk).

#### Materials availability

This study did not generate new unique reagents.

### Experimental model and study participant details

#### Cell lines

T-REx-293 (Tetracycline Repressor Protein expression cells, Invitrogen™, R71007) and HEK-293T cells (Human Embryonic Kidney cells, ATCC, CRL-1573) were used to produce Ad5 and SARS-2 PV virus stocks, respectively. Original CHO cell lines were obtained from ATCC (CCL-61). The CHO-CAR (Chinese Hamster Ovarian cells, transfected to express Human CAR)^42^ and CHO-ACE2-TMPRSS2 (Chinese Hamster Ovarian cells, expressing Human ACE2 and TMPRSS2)stable cell lines were used in transduction assays with Ad5.GFP and SARS-2 PV, respectively. The CHO-ACE2-TMPRSS2 stable cell line was generated using the MT126 pRRL- SFFV-ACE2-IRES (AddGene, 145839) and MT131 pRRL- SFFV-TMPRSS2.v1-IRES (AddGene, 145843) plasmids.^43^ T-REx-293 and HEK-293T cells were cultured in DMEM media (Dulbecco’s Modified Eagle’s Medium; Sigma-Aldrich, Gillingham, UK #D5796), whilst CHO-CAR and CHO-ACE2-TMPRSS2 cells were cultured in DMEM-F12 media (Dulbecco’s Modified Eagle’s Medium/Nutrient Mixture F-12 Ham; Sigma-Aldrich, Gillingham, UK #D0697). All media were supplemented with 10% FBS (Foetal Bovine Serum; Gibco, Paisley, UK #10500-064), 2% Penicillin and Streptomycin (Gibco, Paisley, UK #15070-063) and 1% L-Glutamine (stock 200 mM; Gibco, Paisley, UK #25030-024). CHO-ACE2-TMPRSS2 cells were also passaged with 2μg/mL Puromycin and 100μg/mL Hygromycin once a week. Cells were grown at 37°C with 5% CO_2_. Dulbecco's Phosphate Buffered Saline (PBS, Gibco™, #10010023) and 0.05% Trypsin (Gibco™, #11590626) were used for subculture.

### Method details

#### Virus production

Ad5 was modified to express Green Fluorescent Protein (GFP)^40^ and was propagated in T-REx-293 cells expressing E1 gene products and purified using Caesium Chloride gradient ultracentrifugation as previously described.^45^ Stock titres were determined by Micro-BCA assay (Pierce, Thermo Fisher, Loughborough, #23235), assuming that 1μg protein was equal to 4 x 10^9^ virus particles (vp) and monodispersity was confirmed by Nanoparticle Tracking Analysis (NanoSight NS300, Malvern, UK), which identified the mean diameter of particles in the stock solutions. Infectious titres were quantified by end-point dilution plaque assay, performed in T-REx-293 cells, determining plaque forming units per millilitre (PFU/ml).

The SARS-CoV-2 Pseudotyped Lentivirus (SARS-2 PV) contained a HIV core and expressed Wuhan strain SARS-CoV-2 Spike Proteins (GenBank accession: 43740568) on their viral envelope. SARS-2 PV are replication deficient and express GFP under the control of a spleen focus-forming virus (SFFV) promoter post transduction.^46^^,^^47^ SARS-2 PV were produced in HEK-293T/17 cells (ATCC CRL11268) that were pre-seeded in a T175 flask (Thermo) with approximately 5 x10^6^ cells the day before transfection. Cells were then co-transfected with 2 μg of packaging lentiviral core p8.91,^44^ 3 μg of pCSGW encoding Green Fluorescent Protein,^44^ and 2 μg of the spike SARS2 (D614G)-pCAGGS (Medicines & Healthcare Products Regulatory Agency, #CFAR100985) using FuGENE HD (Promega, UK, #E2311) transfection reagent at a ratio of 1:3 DNA:Fugene in optiMEM (Gibco, Thermo, UK, #31985062). SARS-2 PV were harvested at 48h post transfection and supernatant filtered through a 0.45 μm acetate cellulose filter (Starlab, Milton Keynes, #E4780-1453).^41^^,^^48^ Functional titres were determined by plaque assay.

#### Experimental setup of the closed-system model

The standard closed-system model (Figure 1) was optimised and altered for some experiments, however the general setup remained consistent in each run. A medical grade nebuliser (Aerogen® Solo Starter Kit, Aerogen Ltd, Galway, AG-A53000-XX) was used to aerosolise 10ml of each sample into a 3L reaction kettle (QuickFit™ Wide Neck Flask Reaction 3L, Scientific Laboratory Supplies Ltd, UK, QFR3LF). The nebuliser emitted droplet sizes of 4.47 ± 0.05 μm, at an aerosol output rate of 0.536 ± 0.01 ml/min, as determined by laser diffraction (Spraytec; Malvern Panalytical Instruments).^49^ Aerosolised samples containing virus therefore consisted of 4.47 ± 0.05 μm sized media droplets, each containing a dispersion of virus particles (each approximately 90-100nm in diameter).The reaction kettle was fitted with a lid containing multiple culture vessels (QuickFit™ Borosilicate Glass Flange Lid, Fisher Scientific, Leicestershire, MAF3/52), enabling the insertion of samples and materials, whilst maintaining an air-tight system. Ultravision™ technology was used to induce electrostatic precipitation. The power supply (Ultravision™ Generator, BOWA Medial UK, Newton Abbot, DAD-001-015) was stationed outside of the closed system. The discharge electrode (Ionwand™, BOWA Medial UK, Newton Abbot, DAD-001-003) was inserted into the reaction kettle through a Suba-Seal®, 15cm from the bottom of the reaction kettle and 7cm from either side of the reaction kettle. The power supply was attached to copper tape that covered the inside of the reaction kettle via a modified patient return electrode cable, functioning as a positively charged collector-plate. It is important to note that copper ions are virucidal, and therefore may affect viral viability. As a countercheck, an experimental run was performed using biologically inert stainless-steel as the positively charged collector-plate, to determine whether copper affected the viability of electrostatically precipitated viral particles. Stopcock adapters (QuickFit™ Borosilicate Glass Stopcock Adaptors with Sockets, Fisher Scientific, Leicestershire, MF14/3/SC) were placed throughout the system, ensuring unidirectional flow of the aerosol. A vacuum unit (Duet Flat- Back Aspirator, SSCOR, US, 2314B) was used, at maximum flow rate (>30LPM), to suction the aerosol through the reaction kettle and into a sampling system (BioSampler®, SKC Ltd, Dorset, 225-9595). The sampling system (assembled as per manufacturer’s instructions) contained 2ml sterile serum-free media (DMEM) to recover the captured aerosol samples. To prevent viral contamination, a cold-trap (QuickFit™ Cold-trap, VWR, Pennsylvania, 201-3052) was fitted between the sampling system and the vacuum unit. All experimentation was conducted in a Class II laminar flow hood, and all materials were autoclaved or sterilised with 70% Industrialised Methylated Spirit (IMS) (Thermo Fisher, #15950957, Leicestershire) before and after use.

#### Experimental procedure

To mimic the release of bioaerosols that occurs during key-hole surgery, we developed a closed-system model representing laparoscopy within a peritoneal cavity. A 3L reaction kettle was used to resemble the peritoneal cavity, which is sufflated to approximately 3L with CO_2_ during laparoscopy.^42^ The discharge electrode was positioned within the reaction kettle, directly above the region of bioaerosol release, as it would be during laparoscopy. Quick-fit® glassware was used to ensure that the entire model was air-tight, preventing the release of virally contaminated aerosols.

In each experimental run, 10ml samples were aerosolised into the reaction kettle, which was heated to 37°C to avoid sample condensation and to resemble the average Human body temperature. Closed surgeries using electrocautery devices produce particle sizes of 0.07μm, whilst Ultrasonic scalpels produce particle sizes between 0.35-6.5μm.^50^^,^^51^ Particles produced by the nebuliser were approximately 4.5μm in size, and virus particles (90-100nm diameter) were dispersed within each particle, thus resembling aerosol particles that are released during surgery. The samples were exposed to inactivate/active EP, until the entire sample had been completely aerosolised (1 hour/sample). Samples aerosolised through the system included: Serum-free media (negative control), Ad5.GFP diluted to 1 x 10^10^vp/ml in media and SARS-2 PV diluted to 1 x 10^7^pfu/ml in media. Both viruses expressed GFP for detection in experimental assays. Additionally, 2ml of each sample was not aerosolised through the system (‘non-exposed’) and was immediately stored at -80°C to be used as ‘untreated’ controls. A vacuum unit was employed to suction the aerosol through the closed-system model in a unidirectional flow into the sampling system for sample recovery, to assess viral presence within the aerosol following exposure to EP. Recovered samples were analysed for viral presence by qPCR and for viral activity via transduction and plaque assays. Immediately after complete sample aerosolisation, the collected samples were stored at -80°C. Physical parameters thought to affect the efficiency of EP were altered, in an attempt to determine optimal EP settings. Such parameters included temperature, voltage, the number of discharge electrodes within the reaction kettle and the material of the collector plate attached to the positively charged return electrode.

#### Quantification of viral genomes by qPCR

DNA was extracted using the QIAamp MinElute Virus Kit (Qiagen, USA, #57704). Purified DNA was eluted in 50μl of Ultra-Pure Water (UltraPure™ DNase/RNase-Free Distilled Water, Invitrogen™, Thermo Fisher, #11538646) and stored at -20°C. DNA extracted from the virus stocks were used as standards (Serial dilution: undiluted (200ng/μl), 10^-1^, 10^-2^, 10^-3^, 10^-4^, 10^-5^ and 10^-6^). DNA extracted from experimental samples remained undiluted. Primers (Ad5 Hexon Forward: CCTGCTTACCCCCAACGAGTTTGA, Ad5 Hexon Reverse: GGAGTACATGCGGTCCTTGTAGCTC; P24 Capsid: Forward: GGCTTTCAGCCCAGAAGTGATACC, P24 Capsid Reverse: GGGTCCTCCTACTCCCTGACATG) were used at 10Mm. qPCR for viral DNA was performed using the SYBR Green Master Mix (PowerUp™ SYBR™ Green Master Mix, Applied Biosystems™, Thermo Fisher, #A25741) (per reaction: 15μl Master Mix and 5μl DNA). Reactions were performed in triplicate (for both samples and standards). QuantStudio™ software was used to set the thermal cycling conditions of the qPCR (Pharmaceutical Analytics QuantStudio™ 5 Real-Time PCR System, Applied Biosystems™, Thermo Fisher, #A31670). Samples were held at 50°C for 2 min, followed by 95°C for 2 min. Samples were then cycled at 95°C for 15 sec and 60°C for 1 min for 40 cycles.

#### Transduction assays

CHO-CAR/CHO-ACE2-TMPRSS2 cells were seeded into a 96-well plate at a density of 2x10^4^ cells/well in 200μl complete media and cultured overnight. The following day, complete media was removed, cells were washed briefly in PBS, and experimental samples were added to the cells (100μl, undiluted) and incubated at 37°C for 3 hours. The media was then removed and discarded, and the cells were washed twice with 100μl PBS, prior to replenishing the cells with 200μl total media and culturing for an additional 48 hours. Cells were visualised for GFP expression using a microscopic imaging system (EVOS M7000, Invitrogen™, Thermo Fisher Scientific, #AMF7000), then harvested in FACS buffer and fixed with 4% Paraformaldehyde. Flow Cytometry was performed, using the Accuri (Accuri C6 v.1.0.264.21, BD Biosciences) and the FL1-A channel, to detect virally transduced cells. FlowJo™v10 software was used to analyse all Flow Cytometry data.

#### Plaque assays

T-REx-293/HEK-293T cells were seeded in 12-well plates in complete media, at a density of 1x10^5^ cells/well in triplicate. Cells were cultured for 24 hours, prior to the experiments. Medium was removed, and the cells were washed with 1ml PBS. Experimental samples were added to the wells (1ml, undiluted) in duplicate. The cells were incubated at 37°C for 2 hours, then the medium was removed and replaced with 1ml complete media. The cells were cultured for a further 48 hours, before analysis. Microscopy (EVOS M7000, Invitrogen™, Thermo Fisher Scientific, #AMF7000) was used to image the cells (Objective Lens X20). Transduced cells fluoresced green light under the GFP light source, enabling manual counting of infected cells. The PFU/ml of each sample was calculated using the formula:

$$\left(\frac{\left(Average\;number\;of\;florescent\;\frac{cells}{well}\times 594\left(\frac{Fields}{well}\right)\right)}{\left(Volume\;of\;viral\;sample\;\mu \times dilution\;factor\right)}\right)\times 1000=\frac{PFU}{ml}$$

### Quantification and statistical analysis

All data presented show the mean ± SD. GraphPad Prism v4.03 (GraphPad Software Inc., La Jolla, CA) was used to produce all bar chart figures. The GraphPad Quickcalcs t-test calculator was used to perform the two-tailed paired t-test**.** p-Values of ∗ = p<0.05, ∗∗ = p<0.005, ∗∗∗ = p<0.0005, ns = not statistically significant, p>0.05. All statistical details of the experiments can be found in the figures and figure legends of the results section. The n value is equal to the number of technical repeats.

## Data Availability

• All flow cytometry data presented in this study are deposited in the Mendeley data repository (FCS files) and are publicly available as of the date of publication. All qPCR data presented in this study are deposited in the Mendeley data repository (EDS/EDT files) and are publicly available as of the date of publication. Accession numbers are listed in the key resources table. • This paper does not report original code. • Any additional information required to reanalyse the data reported in this paper is available from the lead contact upon request.
