# Supplementary material for: Capture and inactivation of viral particles from bioaerosols by electrostatic precipitation
Source: iScience. 2023 Aug 9;26(9):107567. doi: 10.1016/j.isci.2023.107567 (PMC10470311; doi:10.1016/j.isci.2023.107567)
Supplement: Document S1. Figures S1–S6 [file mmc1.pdf]

iScience, Volume *26*

Supplemental information

Capture and inactivation of viral particles

from bioaerosols by electrostatic precipitation

Hannah E. Preston, Rebecca Bayliss, Nigel Temperton, Martin Mayora Neto, Jason Brewer, and Alan L. Parker

Inactivation of Virus in Bioaerosols using Electrostatic Precipitation.

Supplementary:

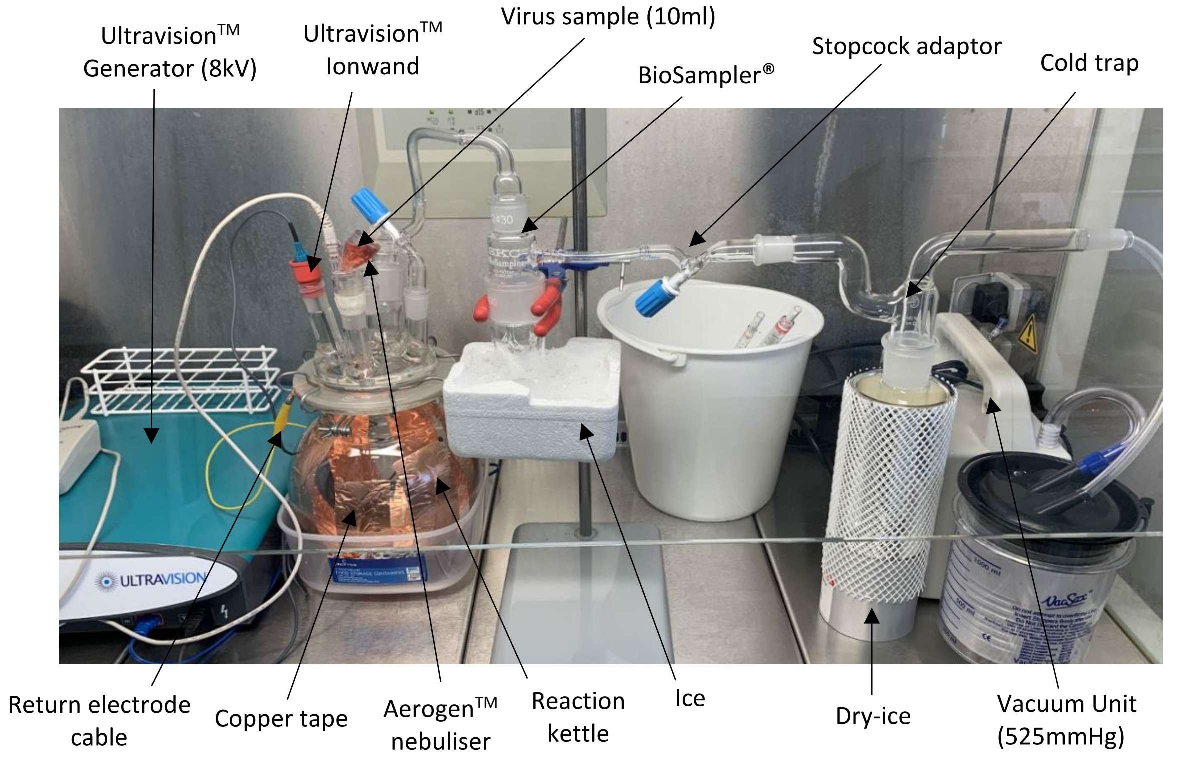

Supplementary Figure 1. Experimental Setup of the Refined Closed-System Model (shown schematically in **Figure 1**). All samples were aerosolised into the air-tight reaction kettle, exposed to Ultravision^{TM} (active/inactive) and suctioned into the BioSampler for recovery and collection. Collected samples were stored at -80°C immediately after each experimental run, prior to experimental analysis.

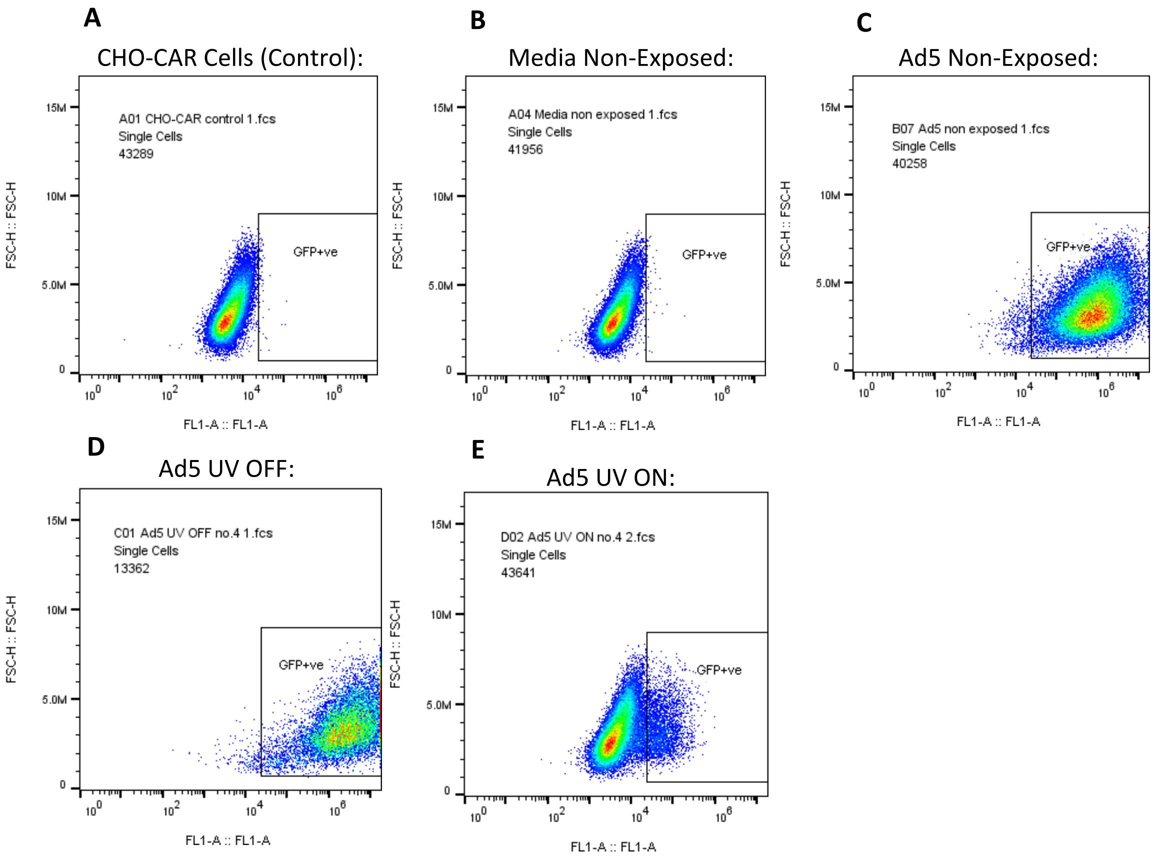

Supplementary Figure 2. Transduction assay raw data (**Figure 2.B**). Cell populations infected with experimental samples analysed by Flow Cytometry and gated with the FL1-A channel, to detect transduced (GFP positive) cells.

Inactivation of Virus in Bioaerosols using Electrostatic Precipitation.

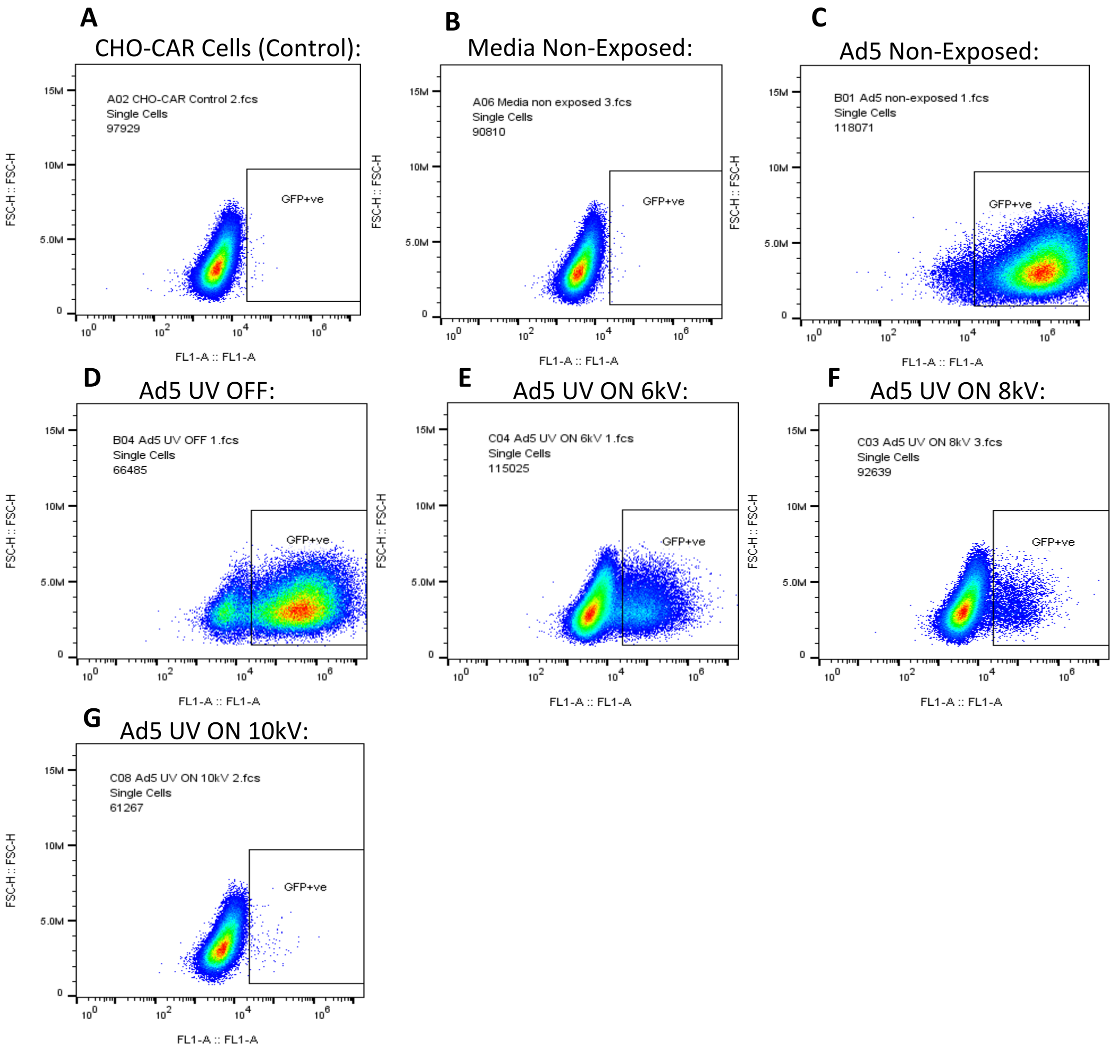

Supplementary Figure 3. Transduction assay raw data (**Figure 3.B**). Cell populations infected with experimental samples analysed by Flow Cytometry and gated with the FL1-A channel, to detect transduced (GFP positive) cells.

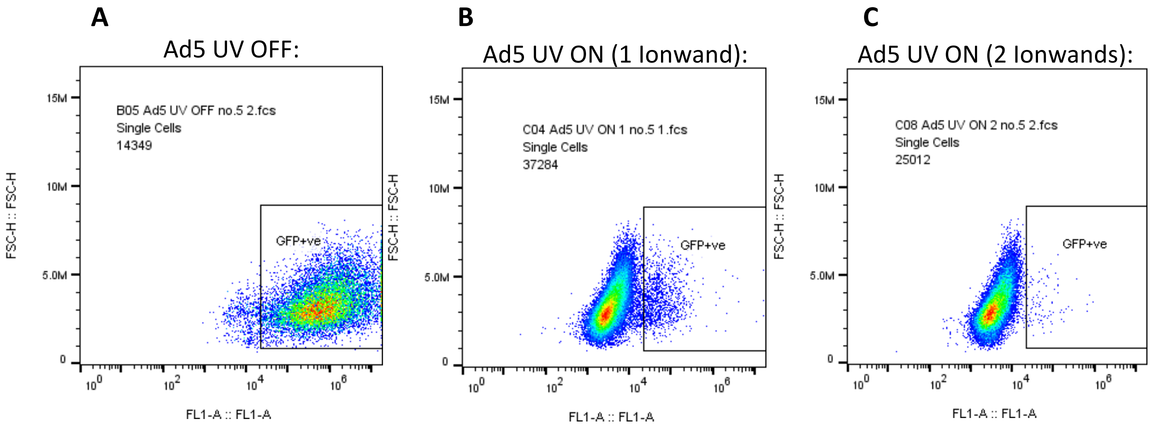

Supplementary Figure 4. Transduction assay raw data (**Figure 4**.**B**). Cell populations infected with experimental samples analysed by Flow Cytometry and gated with the FL1-A channel, to detect transduced (GFP positive) cells.

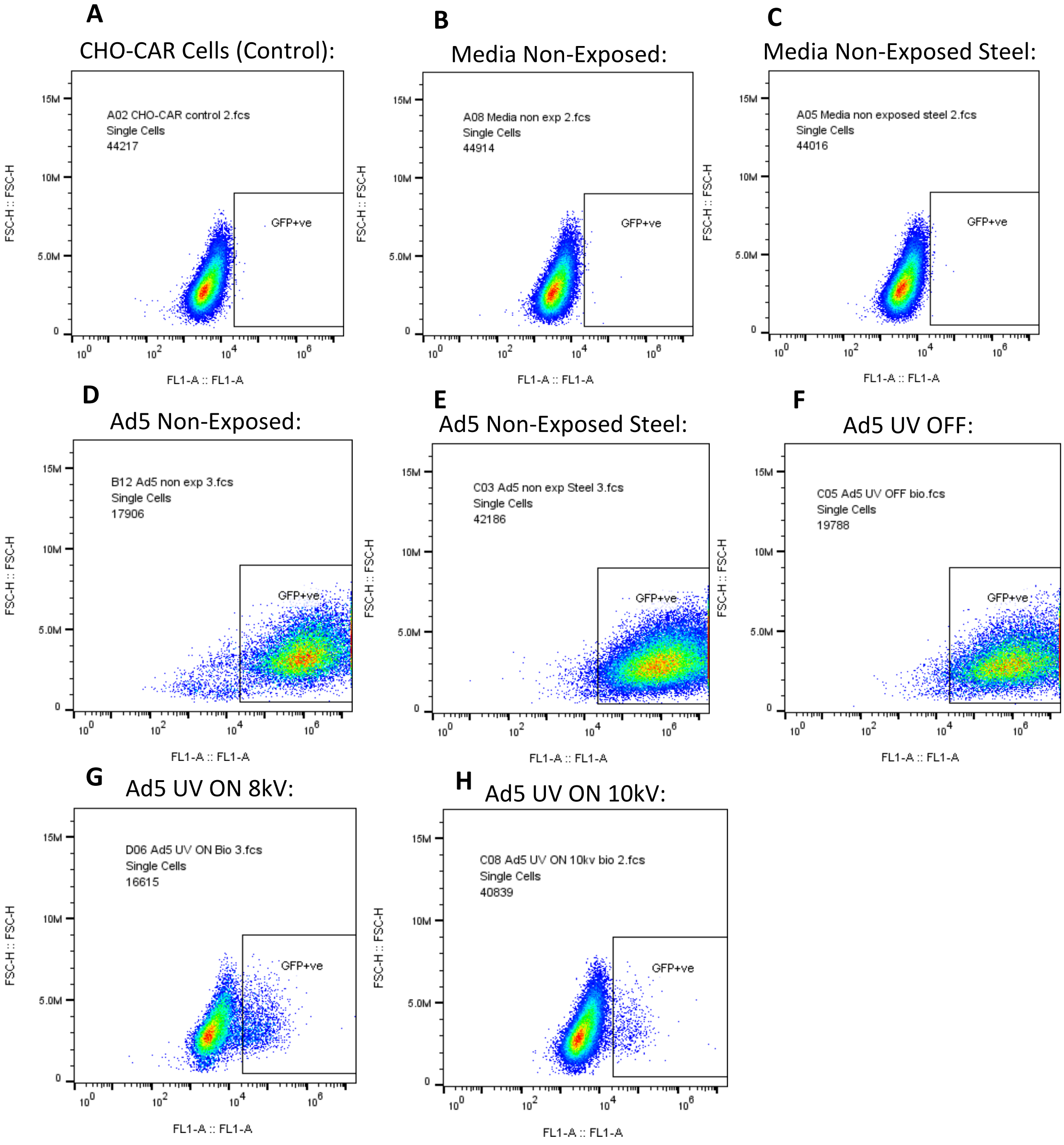

Supplementary Figure 5. Transduction assay raw data (**Figure 5.B**). Cell populations infected with experimental samples analysed by Flow Cytometry and gated with the FL1-A channel, to detect transduced (GFP positive) cells.

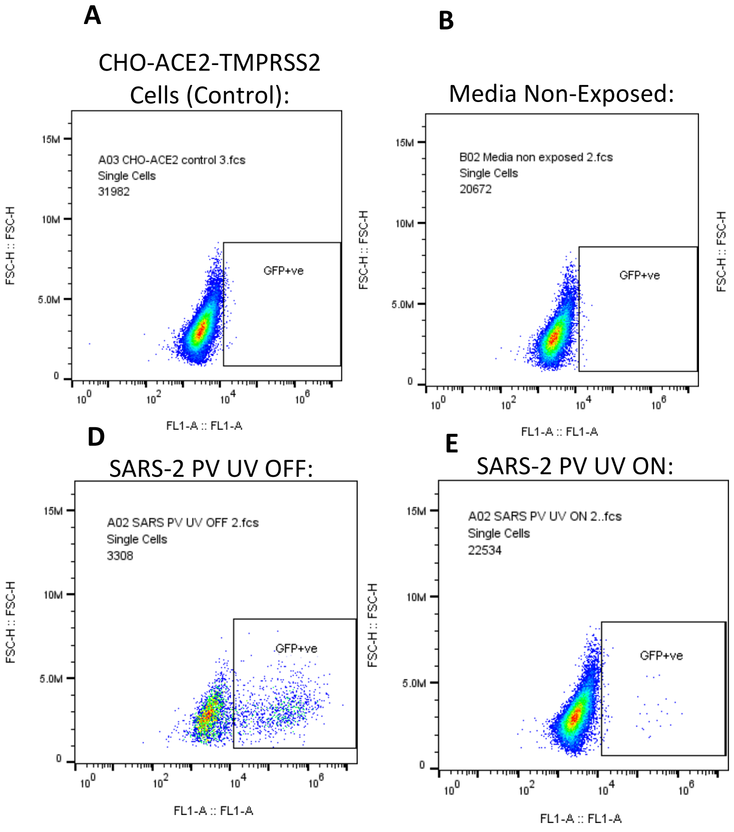

Supplementary Figure 6. Transduction assay raw data (**Figure 6.B**). Cell populations infected with experimental samples analysed by Flow Cytometry and gated with the FL1-A channel, to detect transduced (GFP positive) cells.
